# Effects of a Problem Drinking Prevention Program Developed Based on Bandura’s Self-Efficacy Theory in Nursing Students

**Published:** 2020-01

**Authors:** Jung-Hwa KIM, Young-Ran KWEON

**Affiliations:** 1 Gwangju Buk-Gu Addiction Management Center, Gwangju, Korea; 2 Department of Nursing, Chonnam National University, Gwangju, Korea

## Dear Editor-in-Chief

Problem drinking is considered a serious health-threatening problem in college students. Compared to nondrinking students, college students who drink experience drinking-related issues, such as physical, mental, and academic problems (1).

The best health-promoting activity to reduce problem drinking in college students is prevention education. In particular, providing problem-drinking prevention education and early intervention during the freshman year can increase students’ awareness of the harm caused by alcohol use and curtail problem drinking (2). Delivering accurate knowledge about problem drinking is important for preventing problem drinking. Another key factor in problem-drinking prevention involves increasing drinking refusal self-efficacy (3).

Self-efficacy is a strong predictor of health-related behavior, and drinking refusal self-efficacy is based on Bandura’s self-efficacy theory (4). Teaching healthcare professionals to understand their drinking problems and acquire coping skills helps them to prevent their health-related problems and those of others, and provide more professional help (5). Particularly, nurses play a crucial role in promoting patients’ and community members’ health as health educators. Patients tend to benchmark nurses’ health-related behavior and compare it with their own. For these reasons, nursing students should undergo problem-drinking prevention education to understand their own drinking behavior before beginning clinical training (6).

Therefore, this study aimed to examine the effects of a problem-drinking prevention program, based on Bandura’s self-efficacy theory on drinking-related knowledge, drinking refusal self-efficacy, and drinking behavior, in nursing students.

Bandura (4) proposed the following four resources to increase self-efficacy: performance accomplishment, vicarious experience, verbal persuasion, and emotional arousal. Self-efficacy is boosted and health behavior is altered positively once these four resources are used efficiently (Table 1). The participants were 48 undergraduate nursing students in the first grade, recruited from a University in Gwangju city. The experimental group (n= 25) received a 6-session (60 min/session) problem drinking prevention program based on Bandura’s self-efficacy theory from Oct 15 to Dec 1, 2017, while the control group (n= 23) received the program after the experiment. Data were collected, via self-report structured questionnaires administered before and after the intervention. The study was approved by the institutional review board at Hospitaller Order of St. John of God, Korea (NO. IRB-2017-8).

**Table 1:** Problem Drinking Prevention Program based on Bandura’s Self-Efficacy Theory

***Session***	***Themes***	***Contents***	***Resources of self-efficacy***
1	Understanding of addiction	·Tree of addiction	VP, VE
		·Understanding of neuroscience-based addiction	
2	Awareness of drinking in freshman	·Discussion	VP, VE
		- Advantages and disadvantages of drinking and drinking in college students	
		·Virtual Drinking Experience	
3	Exploring drinking factors to refuse to drink	· Who and how:	VP, VE, EA, PA
		Explore drinking situations	
		· Negative emotions seeking to induce drinking	
		· Exploring interpersonal factors leading to drinking	
4	Drinking refusal training	· Explore high-risk drinking situations	VP, EA
		· Role play for drinking refusal	
5	Drinking refusal training	· Alternative activities: Find fun activities without drinking	VP, VE, PA
		· Explore nearby resources	
6	Setting goals for drinking	· Raising the goal of drinking	VP, VE
		· Evaluation and finishing	
		· Post inspection	

Note: VP=verbal persuasion, VE=vicarious experience, EA=emotional arousal, PA=performance accomplishment

Informed consent was obtained from each student prior to their participation in the study. The data were analyzed with SPSS 21.0 (Chicago, IL, USA), used to calculate the means and standard deviations. Differences in major variables between the two groups were analyzed using an independent t-test. Statistical significance was set at *P*<0.05. After the intervention, undergraduate nursing students in the experimental group reported significant positive changes in drinking-related knowledge (*t*=4.318, *P*<0.001), drinking refusal self-efficacy (*t*=2.195, *P*=0.033), and drinking behavior (*t*= −2.314, *P*=0.022) relative to the control group (Table 2).

**Table 2:** Comparison of Drinking Knowledge, Drinking Refusal Self-efficacy, and Drinking Behavior between Two Groups (N = 48)

***Variable***		***Pre***	***Post***	***Difference (Post-Pre)***	**t**	**P**
		M ± SD	M ± SD	M ± SD		
Drinking knowledge	Exp.	5.76 ± 1.48	7.64 ± 1.38	1.88 ± 1.42	4.318	<0.001
	Con.	5.09 ± 1.59	5.04 ± 1.36	−0.04 ± 1.66		
Drinking refusal self-efficacy	Exp.	22.84 ± 5.27	26.04 ± 5.10	3.20 ± 2.74	2.195	0.033
	Con.	24.87 ± 4.53	25.48 ± 5.80	0.61 ± 5.18		
Drinking behavior	Exp.	6.12 ± 3.26	4.16 ± 2.30	−1.96 ± 1.92	−2.314	0.022
	Con.	6.00 ± 2.91	5.17 ± 2.37	−0.83 ± 1.30		

Note: Exp. = experimental group; cont. = control group, M=mean, SD=standard deviation

The program was effective in preventing problem drinking in nursing students. Therefore, subsequent studies should determine whether this program exerts similar effects on students in other departments. Further, future studies should use and examine the effects of such programs as a strategy for preventing drinking problems early in the freshman year.
